# Lifetime-preserving reference models for characterizing spreading dynamics on temporal networks

**DOI:** 10.1038/s41598-017-18450-3

**Published:** 2018-01-15

**Authors:** Mingwu Li, Vikyath D. Rao, Tim Gernat, Harry Dankowicz

**Affiliations:** 1 Department of Mechanical Science and Engineering, University of Illinois at Urbana-Champaign, Urbana, Illinois USA; 2 Carl R. Woese Institute for Genomic Biology, University of Illinois at Urbana-Champaign, Urbana, Illinois USA; 3 Department of Physics, University of Illinois at Urbana-Champaign, Urbana, Illinois USA; 4 Department of Computer Science, Faculty of Mathematics and Computer Science, University of Leipzig, Leipzig, Germany

## Abstract

To study how a certain network feature affects processes occurring on a temporal network, one often compares properties of the original network against those of a randomized reference model that lacks the feature in question. The randomly permuted times (PT) reference model is widely used to probe how temporal features affect spreading dynamics on temporal networks. However, PT implicitly assumes that edges and nodes are continuously active during the network sampling period – an assumption that does not always hold in real networks. We systematically analyze a recently-proposed restriction of PT that preserves node lifetimes (PTN), and a similar restriction (PTE) that also preserves edge lifetimes. We use PT, PTN, and PTE to characterize spreading dynamics on (i) synthetic networks with heterogeneous edge lifespans and tunable burstiness, and (ii) four real-world networks, including two in which nodes enter and leave the network dynamically. We find that predictions of spreading speed can change considerably with the choice of reference model. Moreover, the degree of disparity in the predictions reflects the extent of node/edge turnover, highlighting the importance of using lifetime-preserving reference models when nodes or edges are not continuously present in the network.

## Introduction

Networks provide a conceptual framework for the study of a wide range of complex systems, from ecosystems and societies^1^ to specific biological subsystems such as the brain^2^. The constituent parts of the system under consideration are represented as nodes of the network, and interactions are represented by edges (also known as links) between nodes.

The simplest version of a network is one with a static topology that captures the connectivity among the different nodes, but ignores the temporal nature of any interactions. Such a description can yield insights into how the network is formed or which nodes are central to its functioning^3^. For instance, many networks, including the internet and protein-protein interaction networks, exhibit scale-free topological properties, suggesting that they are formed by processes of preferential attachment^4^.

On the other hand, one is often interested not only in the structure *of the network*, but also in the dynamics of processes occurring *on the network*. Because connections between nodes are not always active in real networks, it becomes important to add an additional time dimension so that the order of events is preserved in the network representation^5,6^. In the resultant *temporal networks*, the times when edges are active are included as explicit elements in the network representation. Many real-world networks have been modeled as temporal networks, including human and animal proximity networks^7–10^, brain networks^11,12^, economic networks^13^, telephone communication networks^14^, and transportation networks^15^. Analysis of such temporal representations have yielded important insights (reviewed in refs^6,14^).

Conceptually, temporal networks can be viewed in two complementary ways. In the *link picture*, one associates with each edge a sequence of contacts for that edge. The first and last contacts in such a sequence represent the birth (or activation) and death (or deactivation) of the associated edge, and together specify the *edge lifetime*. The duration between these two contacts is the *lifespan of the edge*^16,17^, and the time gaps between subsequent edge contacts are the *edge interevent times* (edge IETs). On the other hand, in the *node picture*, one associates with each node a sequence of contacts for that node – each contact represents an interaction of that node with some other node in the network. The *node lifetime* is specified by both the first contact in this sequence (representing node activation) and the last (representing deactivation). The duration between these two contacts is the *lifespan of the node*, and the time gaps between subsequent node contacts are the *node interevent times* (node IETs).

Temporal structures in real networks can be characterized using the elementary concepts described in the previous paragraph. An important example is *burstiness*, characterized by the presence of a broad, heavy-tailed distribution of edge/node IETs. Burstiness appears to be a universal feature of real-world communication interactions^5^. Another temporal structure that has recently attracted attention is the distribution of edge lifespans. A broad distribution of lifespans reflects the fact that edges are not continuously active^18,19^. A related feature is the heterogeneous distribution of edge birth times^17,18^. Notably, nodes can be continuously active^17^ with constant activity rate^20^ even as the associated edges undergo birth-death processes.

Temporal structures such as burstiness and heterogeneous node or edge lifespans can have strong effects on the dynamics of processes occurring on temporal networks. Spreading is one such process that occurs in various guises in real-world networks, from disease propagation in animal social networks to viral or rumour spreading in electronic communication networks. Understanding the effects of different temporal structures on spreading dynamics is an important first step in understanding and manipulating such dynamics, whether for the purpose of containing the spread of an epidemic through face-to-face contacts or for enhancing the spread of information through a communication network.

Over the past decade, many efforts have been made to understand the influence of burstiness on spreading; in empirical settings^9,21,22^, in synthetic networks^23^ and in theoretical models^24,25^. Observations on real-world networks suggest that burstiness co-occurs with an apparent slow-down of spreading, the known exceptions being a network of contacts between sex sellers and buyers^22^ and recent observations on honey bee social networks (Gernat *et al*., under review (2017)). A fully-developed theoretical understanding of the relationship between burstiness and spreading is not yet available, although it is clear that burstiness is not the only factor influencing spreading dynamics. For example, it has been shown that even in the presence of burstiness, the speed of spreading can be modulated by the network topology or the specific form of the IET distribution^25–27^.

To date, efforts devoted to understanding how spreading is affected by the lifespan of edges and nodes are limited^6^. Holme and Liljeros^16^ have demonstrated that the birth and death of links can modulate the occurrence of epidemic outbreaks in epidemiological models, although their work did not study the influence of node lifespan on spreading dynamics. Using synthetic models, Rocha and Blondel^23^ have also shown that higher node turnover rates can increase the prevalence of infected nodes in epidemic spreading.

Randomization techniques are powerful tools for studying the effects of a temporal structure (such as burstiness) on spreading on an empirical network. To understand the effect of a given structure, one seeks to compare measurements (such as simulated mean prevalence at a fixed time) made on the network against the same measurements in randomized versions of the network that modify the structure in question.

One popular reference model is the randomly permuted times (PT) model^5,6^. Briefly, PT reshuffles timestamps across all edges in a temporal network. It thus preserves the topological structure of the network, as well as some temporal features (such as daily patterns of activity), but modifies others, including the order of events, burstiness, the mean IET of an edge, edge activation times, deactivation times, and lifespans of edges and nodes. While the modification of the order of events is deliberate and enables one to study its effect on spreading dynamics^5,6^, concomitant changes to other temporal features may be unintentional but can still affect spreading properties of the reference network.

For instance, if the lifespan of a typical edge in a temporal network is much smaller than the observation time *T*, then applying PT typically results in a reference network with a higher mean lifespan across edges. This may then change the speed of spreading on the reference network, making it difficult to disentangle the effect of the lifespan distribution from that of other temporal features, such as burstiness. For networks with nodes entering and leaving dynamically, PT can increase the mean lifespan of nodes^23^, with similar potential consequences to the analysis. For these reasons, it is especially important to be careful when applying PT to temporal networks with broad distributions of node or edge lifespans (due perhaps to short-lived nodes or edges).

In a forthcoming paper, Gernat *et al*. propose a restricted version of PT (here denoted by PTN), which preserves the lifetimes of nodes. This restriction was developed in order to investigate whether bursty interaction patterns and rapid spreading dynamics co-exist in automatically recorded honey bee social networks in which nodes leave the network dynamically due to mortality. In this paper, we systematically study PTN in four empirical networks, and additionally propose and investigate a further restriction (denoted by PTE) that also preserves edge lifetimes. Specifically, the PTE and PTN reference models preserve the first and last timestamps of contacts on edges and nodes, respectively, while permuting timestamps of contacts that occurred within their lifetime, with the timestamps of other contacts that took place during the lifetime of the edge or node in question. By construction, these reference models therefore preserve the activation times, deactivation times, lifespans, and mean IETs of edges and nodes, respectively. This enables us to investigate the effects of bursty edge IETs and node IETs on spreading *without* the presence of artefacts due to altered edge or node lifetimes.

We use four publicly-available empirical networks, whose spreading properties have been previously analyzed, to illustrate the importance of taking these considerations into account. For each of these networks, we compare the dynamics of spreading on PTE and PTN reference networks with spreading on a PT reference network. In addition, we build artificial networks *in silico* – using a protocol that allows us to tune certain temporal features, such as the heterogeneity of edge lifespans – and study how spreading dynamics on these synthetic networks varies relative to the corresponding PT, PTE, and PTN reference models.

## Results

### Overview

We begin with a brief introduction to the empirical data sets used in our study. We then quantify various temporal structures in these data sets, including burstiness and edge and node lifespans. In particular, we examine node and link turnover dynamics in four empirical networks, and let our observations motivate the introduction of the two structure-preserving reference models in the following subsection. Next, we turn to the analysis of spreading on the empirical networks, and highlight the different predictions that result from these new reference models. Finally, we study spreading dynamics on synthetic networks, in which two specific temporal structures may be explicitly tuned, viz., the exponent of the edge IET distribution and the heterogeneity of edge lifespans.

### Empirical networks

We analyze spreading dynamics on four publicly-available empirical data sets. The first data set (Ant) consists of interactions between ants in a colony^9^. The second data set (Prostitution) is a sexual contact network between escorts and sex buyers, estimated from interactions in a web forum^22^. We also analyze two human, face-to-face proximity data sets gathered using radio-frequency identification sensors. One data set (Conference) describes social interactions of participants at a scientific conference^7^, and the other (Workplace) describes contacts in an office building of the French Institute for Public Health Surveillance^8^. These four data sets were chosen to sample networks with different combinations of features in terms of node turnover and the distribution of edge lifespans. As we will show in the following subsection, the Ant network has both node turnover and a broad edge-lifespan distribution, the Prostitution network has node turnover but low heterogeneity in edge lifespans, and the two face-to-face data sets are well-described in the ongoing node picture and have heterogeneous edge lifespans. Basic network statistics for each of these data sets are listed in Table 1.

**Table 1:** Basic statistics of empirical data sets.

	*N*	*M*	*L*	*T*	Δ*t*	*ρ*	*f*_*N*_	*f*_*E*_
Ant	89	649	1834 (1911)	1438 s	1 s	2.82	0.08	0.57
Prostitution	14783	33875	43906 (44088)	1232 d	1 d	1.30	0.45	0.88
Conference	113	2196	9865 (20818)	212340 s	20 s	4.49	0.02	0.24
Workplace	92	755	4592 (9827)	987620 s	20 s	6.08	0.03	0.19

*N* and *M* are the number of nodes and edges, respectively, in the network aggregated over the entire sampling time. *L* is the number of contacts. *T* and Δ*t* are the sampling time and time resolution of data sets. *ρ* is the average number of contacts per edge (i.e. *ρ* = *L*/*M*). *f*_*N*_ (*f*_*E*_) is the fraction of contacts that correspond to either the activation or deactivation of nodes (edges). Preprocessing the original data sets, as described in the Methods section, reduced the number of contacts per network; the original numbers of contacts are included in parentheses.

### Temporal contact patterns of edges and nodes

We characterize the burstiness of interactions by measuring the burstiness coefficient^28^ for the distribution of node IETs (*B*_*N*_ in Table 2) and edge IETs (*B*_*E*_ in Table 3). Nodes and edges in the Conference and Workplace data sets have higher burstiness than in the other networks. Additionally, the burstiness for node IETs is in general higher than that for edge IETs.

**Table 2:** Burstiness coefficient *B*_*N*_ and mean node IETs 〈Δ_*N*_〉 of nodes in empirical networks and their reference models.

	*B*_*N*_				〈Δ_*N*_〉	
	Empirical	PT	PTE	PTN	Empirical	PT
Ant	0.42	0.25 (0.01)	0.37 (0.01)	0.25 (0.03)	22.7 s	32.9 (0.2) s
Prostitution	0.37	0.24 (0.00)	0.37 (0.00)	0.33 (0.00)	39.8 d	86.4 (0.4) d
Conference	0.71	0.62 (0.00)	0.68 (0.00)	0.65 (0.00)	971 s	1175 (1) s
Workplace	0.63	0.51 (0.00)	0.57 (0.00)	0.53 (0.00)	8373 s	9703 (19) s

We round the values of *B*_*N*_ to 2 decimals. For *B*_*N*_ and 〈Δ_*N*_〉 of reference models, we generate four randomized networks for each case, and list the mean *μ* and standard deviation *σ* of the burstiness in the form *μ*(*σ*) in the table. Note that 〈Δ_*N*_〉 is preserved by the PTN and PTE transformations.

**Table 3:** Burstiness coefficient *B*_*E*_ and mean edge IETs 〈Δ_*E*_〉 of edges in empirical networks and their reference models.

	*B*_*E*_				〈Δ_*E*_〉		
	Empirical	PT	PTE	PTN	Empirical	PT	PTN
Ant	0.28	0.05 (0.01)	0.21 (0.01)	0.05 (0.01)	84.8 s	225.7 (0.7) s	154.3 (1.4) s
Prostitution	0.17	−0.02 (0.00)	0.16 (0.00)	0.12 (0.01)	87.6 d	246.4 (0.7) d	105.9 (0.9) d
Conference	0.47	0.27 (0.01)	0.42 (0.00)	0.31 (0.00)	10445 s	19427 (78) s	16095 (143) s
Workplace	0.48	0.28 (0.00)	0.41 (0.00)	0.31 (0.00)	44765 s	76147 (969) s	64657 (751) s

We obtain results of *B*_*E*_ and 〈Δ_*E*_〉 for reference models in the same way as in Table 2. Note that 〈Δ_*E*_〉 is preserved under PTE.

In order to check whether the burstiness coefficient *B*_*E*_ for edge IETs is a meaningful quantity, we calculate the average number *ρ* of contacts for edges and list the results in Table 1. In the limiting case *ρ* = 1, where each edge has only one interaction, the node IETs are fully determined by the edge activation dynamics of the network^17,18^. We see from the table that in the Prostitution network, *ρ* = 1.3, indicating that most edges have only one interaction within the sampling time window. The applicability of *B*_*E*_ therefore seems limited here, as it only measures bursty edge IETs for the small fraction of edges with multiple contacts.

**Figure 1:**
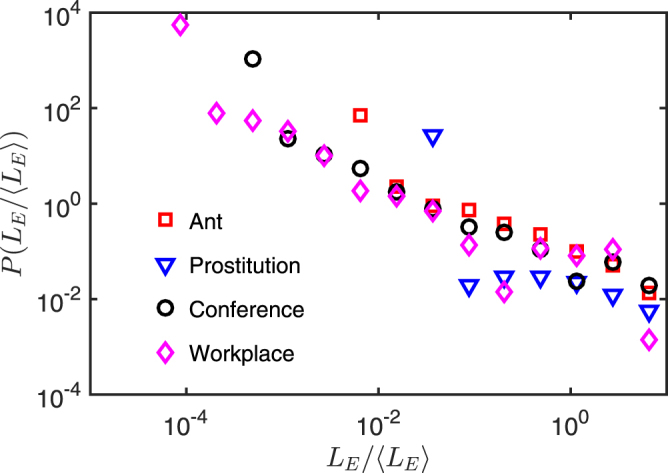
Distribution of lifespans of edges for four temporal networks. *L*_*E*_ is the lifespan of an edge, calculated as the time difference between the timestamps of the last and the first contact of the edge; 〈*L*_*E*_〉 is the mean of *L*_*E*_. Edges with a single contact are assigned a lifespan equal to the sampling resolution. Bin widths are uniform in log space on the interval [10^−5^, 10]. The Prostitution network has a large number of edges with a single contact, resulting in a peak at the left end of the distribution.

Next we present the edge lifespan distributions for the four empirical networks. As can be seen in Fig. 1, the edge lifespans display a broad distribution that spans at least an order of magnitude on the abscissa. This has also been reported in the context of human communication networks^18,19^, suggesting that the edge dynamics are better described by the turnover picture than by the ongoing-link picture^16,17^. We note that the Prostitution data set is less heterogeneous in terms of edge lifespans due to the large number of edges with a single contact.

To check whether the nodes also display turnover dynamics – i.e., whether nodes enter and leave the networks dynamically – we study the distribution of activation/deactivation times and node lifespans. Recall that *T* denotes the sampling time of a temporal network and let *t*^*a*^ and *t*^*d*^ be the activation and deactivation times (relative to the start of sampling). We then consider the distributions of the following normalized quantities measuring the node activation time, deactivation time and lifespan respectively:

1 $${\tau }^{a}=\frac{{t}^{a}}{T};\quad {\tau }^{d}=\frac{{t}^{d}}{T};\quad {\tau }^{ad}=\frac{{t}^{d}-{t}^{a}}{T}.$$

**Figure 2:**
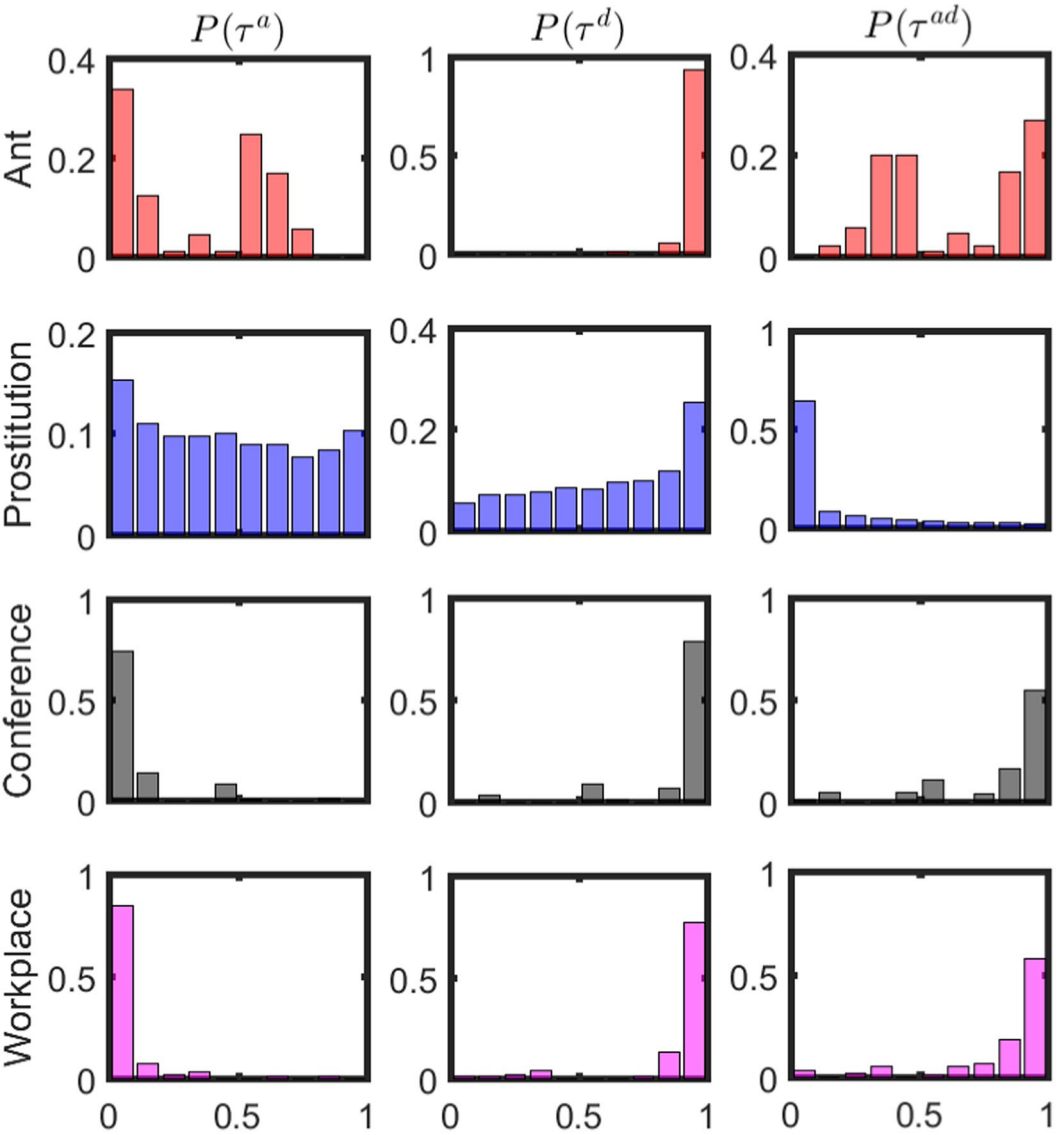
Distributions of activation times, deactivation times, and lifespans of nodes for four empirical networks. The columns present probability density distributions of normalized activation times, deactivation times, and lifespans of nodes, respectively, while rows correspond to results for the Ant, Prostitution, Conference and Workplace networks, respectively. In all cases, bin sizes are set to 0.1.

For networks in which nodes are continuously present and active, we can expect that the density *P*(*τ*^*a*^) will be much larger for small *τ* ^*a*^, while *P*(*τ* ^*d*^) and *P*(*τ*^ *ad*^) will carry the most weight as *τ* ^*d*^, *τ*^*ad*^ → 1. In Fig. 2, we plot the distributions of *τ* ^*a*^, *τ* ^*d*^ and *τ* ^*ad*^ for the four empirical networks. From these plots, we see that whereas most nodes enter near the beginning and leave near the end in the Conference and Workplace networks, in the Ant and Prostitution networks, nodes enter and leave the network during the sampling interval. Thus we conclude that the ongoing-node picture holds in the Conference and Workplace networks, while nodes in the Ant and Prostitution networks exhibit node turnover dynamics.

### Structure-preserving reference models

**Figure 3:**
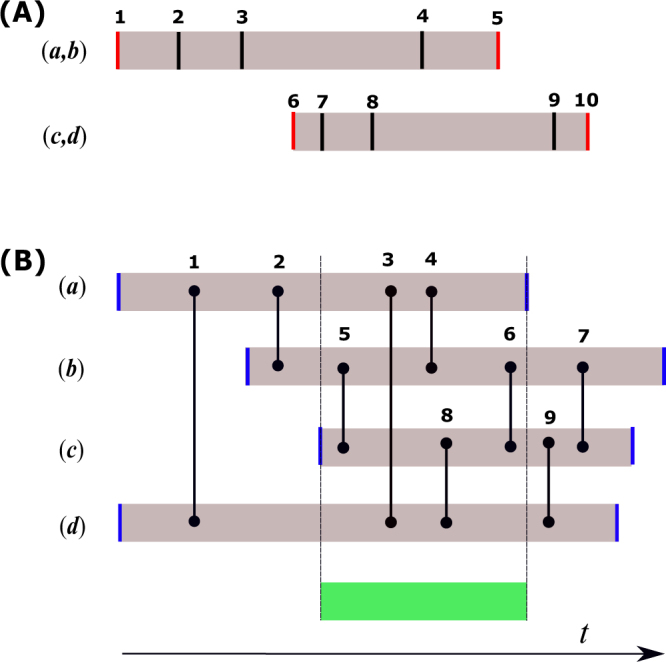
Construction of the PTE and PTN reference models. In (**A**), we illustrate PTE by considering two edges (*a*, *b*) and (*c*, *d*) with 5 contacts each, labelled 1–5 and 6–10 respectively. Vertical lines represent the time when a contact is initiated. Red lines are contact initiation times that “activate” or “deactivate” an edge. We only permute times that do not activate or deactivate an edge. In this example, eligible permutations are (4, 7) and (4, 8). By contrast, (2, 7) is ineligible because the lifespan of (*c*, *d*) would be extended. In (**B**), we illustrate PTN. Vertical lines represent contact initiation times between nodes (*a*, *b*, *c*, *d*). Blue lines are node activation and deactivation times. Shaded areas depict the lifespans of nodes. To permute contact pairs from edges (*a*, *b*) and (*c*, *d*), we first obtain the intersection of the lifespans of nodes *a*, *b*, *c* and *d*, which is highlighted in green. The permutation of contact times is allowed only if the two contacts to be permuted are both located within the green interval. For instance, (4, 8) is an eligible permutation while (2, 9) is rejected because it would extend the lifespans of nodes *a* and *c*.

The PTN reference model was proposed and implemented in Gernat *et al*. to accurately quantify spreading dynamics on honey bee social networks. In their experiments, bees died while the networks were sampled, resulting in node turnover. Here, we analyze PTN in detail and introduce a further modification of PTN that also preserves the lifetime of edges (PTE) The basic idea of PTE (PTN) is to preserve the activation and deactivation times of each edge (node) while performing pairwise permutation operations to randomize contacts in a temporal network. A simple illustration of the method of construction is presented in Fig. 3. We give a detailed description of the construction in the Methods section. Since the activation and deactivation times – and thus the lifespans – of edges/nodes are preserved, the mean edge IETs and the mean node IETs are also preserved. Table 4 lists the subset of temporal structures that are preserved under each of the reference models.

**Table 4:** Temporal structures retained in different reference models.

	${t}_{{\boldsymbol{E}}}^{{\boldsymbol{a}}}$	${t}_{{\boldsymbol{E}}}^{{\boldsymbol{d}}}$	${t}_{{\boldsymbol{N}}}^{{\boldsymbol{a}}}$	${t}_{{\boldsymbol{N}}}^{{\boldsymbol{d}}}$	*L*_*E*_	*L*_*N*_	〈Δ_*E*_〉	〈Δ_*N*_〉	D	*B*
PT									✓	
PTN			✓	✓		✓		✓	✓	
PTE	✓	✓	✓	✓	✓	✓	✓	✓	✓	

${t}_{E}^{a}$: edge activation time; ${t}_{E}^{d}$: edges deactivation time; ${t}_{N}^{a}$: node activation time; ${t}_{N}^{d}$: node deactivation time; *L*_*E*_: edge lifespan; *L*_*N*_: node lifespan; 〈Δ_*E*_〉: mean IET of edges; 〈Δ_*N*_〉: mean IET of nodes; D: daily patterns; B: bursty edge IETs and node IETs.

We illustrate the effects of and differences between PT, PTE, and PTN by calculating the burstiness coefficients, the mean edge IET, and the mean node IET after applying these reference models to the four empirical data sets. Table 2 shows that *B*_*N*_ is reduced after permutation. If we rank the models in ascending order of the extent of reduction, we obtain the ordering: PTE, PTN, and PT. Importantly, PT increases the mean IET of nodes, 〈Δ_*N*_〉, which may lead to slower spreading in the resulting reference networks. PTE and PTN, on the other hand, preserve 〈Δ_*N*_〉. Using Table 3, we again rank the models by the extent to which *B*_*E*_ is reduced and obtain the same order as for *B*_*N*_. Note that the extent of reduction of *B*_*N*_ or *B*_*E*_ under randomization is negatively dependent on the fraction of contacts that are not eligible for permutation in that reference model (cf. *f*_*N*_ and *f*_*E*_ in Table 1). In addition, PT increases the mean IET of edges, 〈Δ_*E*_〉, more than PTN. We note also that *B*_*N*_ is not significantly reduced under PTE in the Prostitution data set, likely a consequence of the fact that most edges have only one interaction (*ρ* = 1.3) and most contacts are preserved under PTE (*f*_*E*_ = 0.88).

### Spreading dynamics on empirical networks

**Figure 4:**
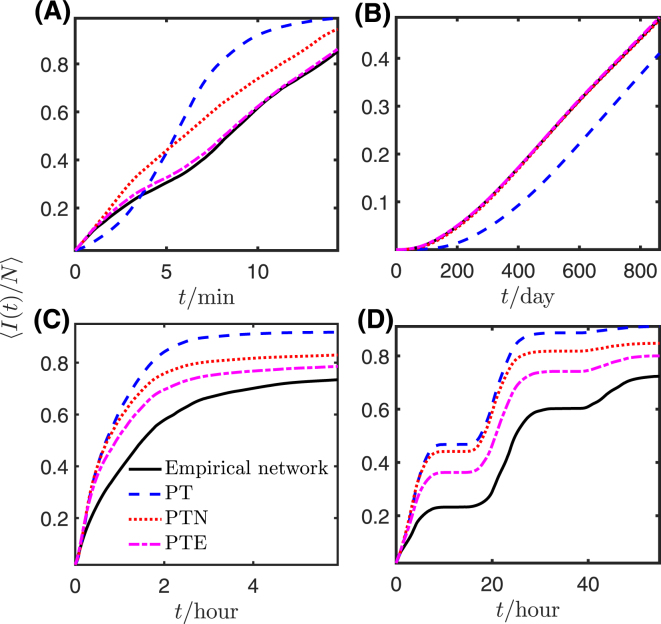
Mean prevalence evolution on empirical networks and the corresponding reference models. Each panel shows the mean fraction of infected nodes, 〈*I*(*t*)/*N*〉, at each point in time for the original contact sequence (black solid line) and the PT, PTN, and PTE reference models (each averaged over four reference networks). (**A**) Ant, (**B**) Prostitution, (**C**) Conference, and (**D**) Workplace. In panel (B), the prevalence curves for the empirical network and the PTN and PTE models are so close as to be indistinguishable.

To study the effects of temporal structures on spreading, we run simulations of the deterministic susceptible-infected (SI) model on the empirical temporal networks and on the corresponding reference networks. For each network, we run 500 simulations, each initialized with a randomly-selected pair of “infected” individuals (see Methods section for details), and obtain the mean prevalence curves shown in Fig. 4. As in ref.^21^, we use the average time to reach 20% prevalence to characterize the speed-up or slow-down of spreading on empirical networks compared with the different reference models. Specifically, suppose that ${t}_{0.2},\,{t}_{0.2}^{PT},\,{t}_{0.2}^{PTN}$ and ${t}_{0.2}^{PTE}$ are the average times to reach 20% prevalence in an empirical network and its PT, PTN and PTE reference models (each averaged over four reference networks). Then we define the relative speed-up of spreading *S*_0.2_ as follows:

2 $${S}_{0.2}^{PT}=\frac{{t}_{0.2}^{PT}-{t}_{0.2}}{{t}_{0.2}};\quad {S}_{0.2}^{PTN}=\frac{{t}_{0.2}^{PTN}-{t}_{0.2}}{{t}_{0.2}};\quad {S}_{0.2}^{PTE}=\frac{{t}_{0.2}^{PTE}-{t}_{0.2}}{{t}_{0.2}}.$$

Thus *S* > 0 indicates that spreading is faster on an empirical network than on the corresponding reference model. In general, we use the terms “speed-up” and “slow-down” to refer to the speed of spreading on an empirical network relative to a reference model (corresponding to *S* > 0 and *S* < 0, respectively).

We first compare the results for PT versus PTN. Table 5 shows that although using the PT reference model on average predicts a speed-up in spreading (${S}_{0.2}^{PT}=0.209$) in the Ant data set, PTN instead on average predicts a slow-down (${S}_{0.2}^{PTN}=-0.116$). Table 5 also shows that the large average speed-up reported in the Prostitution data set under the PT reference model is dramatically reduced when we apply PTN. Thus, for the two networks with node turnover dynamics, the PT reference model yields a large average speed-up in spreading speed, which we attribute to an increase in 〈Δ_*N*_〉 (cf. Table 2). In contrast, for the other two networks (in which most nodes are continuously present throughout the sampling time), Fig. 4 shows similar averaged spreading dynamics for small prevalences on the PTN and PT reference models. For higher prevalences (e.g. 〈*I*(*t*)/*N*〉 > 0.7 in the Conference data set), the dynamics diverge, with faster average spreading on the PT model than on the PTN model. This acceleration may be the result of a high likelihood that a few nodes will be activated early on in the PT model, even though they do not enter the empirical network at the start of sampling (cf. Fig. 2). This would enhance spreading, with an effect that becomes more significant with time. Notice that flat regions in Fig. 4(D) result from daily patterns in the empirical data, which are preserved by all three reference models.

**Table 5:** Measured speed-up of spreading *S*_0.2_ for the four empirical networks relative to different reference models.

	${{\boldsymbol{S}}}_{{\bf{0.2}}}^{{\boldsymbol{PT}}}$	${{\boldsymbol{S}}}_{{\bf{0.2}}}^{{\boldsymbol{PTN}}}$	${{\boldsymbol{S}}}_{{\bf{0.2}}}^{{\boldsymbol{PTE}}}$
Ant	0.210 ± 0.054	−0.256 ± 0.014	−0.116 ± 0.034
Prostitution	0.291 ± 0.021	0.007 ± 0.014	−0.002 ± 0.011
Conference	−0.330 ± 0.020	−0.354 ± 0.026	−0.353 ± 0.036
Workplace	−0.465 ± 0.010	−0.488 ± 0.017	−0.399 ± 0.014

The uncertainty is calculated by propagating the standard error in *t*_0.2_ over the four randomized networks generated for each reference model (see Eq. 2).

Next, we turn our focus to the prevalence curves for the PTE models. From Fig. 4 and Table 5, we see that in all four empirical networks, spreading is slower than the average rate on the corresponding PTE model, suggesting that the burstiness of edge interactions (which are present in the empirical network but absent in all reference networks) might slow down spreading. This slow-down is much smaller in magnitude in the two networks with turnover of nodes (especially the Prostitution data set) than in the other two networks. As explained previously, due to the small average number of interactions per edge and the small fraction of contacts that are available for permutation, the PTE transformation leaves many edges unchanged in the Prostitution network, and so spreading on the PTE reference model is similar to the empirical network.

It is instructive to compare the results of spreading on PTN and PTE models. On average, spreading on the PTN reference models is faster than on the PTE reference models. We associate this with the fact that the PTE transformation preserves more temporal structures than PTN (see Table 4). In particular, PTN modifies the heterogeneous distribution of edge lifespans and the distribution of edge activation times. Thus, faster spreading on the PTN model compared with PTE appears to result from these particular temporal features.

### Spreading dynamics on synthetic networks

To study the effect of a broad distribution of edge lifespans on spreading dynamics, we constructed synthetic networks and studied spreading dynamics on these networks and on the corresponding reference models. The method for constructing synthetic networks is described in detail in the Methods section. Briefly, we first generated a static Erdős-Rényi network *G*(100, 0.06) with 100 nodes and edge density 0.06 (which resulted in 294 edges). Then we assigned active intervals and IETs to the edges using truncated power-law distributions. In our synthetic networks, the parameter *λ* controls the heterogeneity of edge lifespans – specifically, smaller values of *λ* yield a more heterogeneous distribution of lifespans. We control the burstiness of edge IETs by tuning the exponent *β* of the power-law distribution. We consider two cases: (1) *λ* varying from 0.001 to 1 to show the difference between PTE and PT as the heterogeneity of edge lifespans increases; and (2) *β* varying from 0.8 to 2.4 to see how burstiness – controlled by the power-law exponent – affects spreading dynamics.

**Figure 5:**
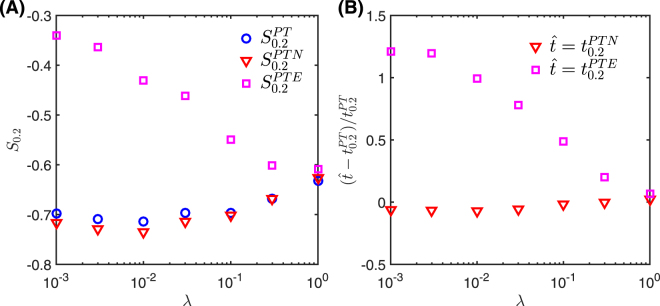
Simulation results for synthetic networks under variations in edge heterogeneity *λ*. Panel (A) shows the average speed-up *S*_0.2_ for the three reference models. In panel (B), we give the relative differences of *t*_0.2_ of PTE and PTN, respectively, with respect to PT.

Figure 5 shows variations in the relative speed-up of spreading as a function of *λ*. For small *λ*, the increased heterogeneity of edge lifespans leads to more edges with few contacts, which in turn diminishes the effect of the PTE randomization simply because permutation is highly constrained for those edges with a small number of contacts. This is reflected in the smaller magnitude of ${S}_{0.2}^{PTE}$ as *λ* decreases. In contrast, PT freely permutes contacts and destroys burstiness across edges regardless of the number of contacts on edges, and is therefore less sensitive to *λ*. When *λ* tends to 1, the relative speed-up of PTE (${S}_{0.2}^{PTE}$) approaches the relative speed-up of PT (${S}_{0.2}^{PT}$).

Although our method of constructing synthetic networks does not directly set the activation/deactivation time of nodes, our simulation results show that most nodes are active during almost the entire sampling time, i.e., that the ongoing-node picture holds in our synthetic networks. Thus, PT and PTN yield similar results, as seen in Fig. 5.

From Fig. 5(A), we see that ${S}_{0.2}^{PT}$ is largely independent of *λ*. While *λ* can adjust the heterogeneity of the lifespans of edges, the burstiness of edge IETs is determined by the bounds Δ_min_ and Δ_max_, and the exponent *β*, of the corresponding truncated power-law probability distribution (see Eq. 4). Since these parameters are independent of *λ*, the burstiness of edge IETs is independent of *λ*, consistent with the independence of ${S}_{0.2}^{PT}$ from *λ*. The difference between ${S}_{0.2}^{PT}$ and ${S}_{0.2}^{PTE}$ can therefore be attributed to the increasing heterogeneity in the edge lifespan distribution as *λ* decreases (cf. Fig. 5(B)).

**Figure 6:**
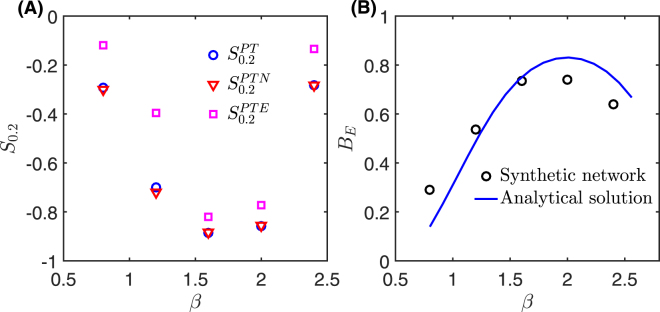
Simulation results for synthetic networks under variations in edge IET power-law distribution exponent *β*. Panel (A) shows the relative average speed-up *S*_0.2_ for the three reference models. Panel (B) shows the burstiness coefficient *B*_*E*_ of edge IETs for the synthetic networks and the analytical solution predicted by the truncated power-law distribution.

Finally, our synthetic networks also allow us to study how the spreading speed varies with the exponent *β* of the edge IET distribution. Figure 6(A) shows the relative speed-up *S*_0.2_ for each of three reference networks as *β* is varied, while Fig. 6(B) shows how the burstiness coefficient *B*_*E*_ of the edge IET distribution varies with *β*. We see that *S*_0.2_ as a function of (*β*) has a minimum between [1.5, 2], while *B*_*E*_ as a function of (*β*) has a maximum in the same range. Thus, it appears that the magnitude of slow-down increases with burstiness, although the value of burstiness does not completely determine the spreading speed. For example, in the rightmost data points in Fig. 6 (at *β* ≈ 2.3), a small decrease in burstiness leads to a large change in *S*_0.2_.

Figure 6(B) includes an analytical prediction of *B*_*E*_ for the truncated power-law probability distribution *P*(Δ; *β*, Δ_min_, Δ_max_) used in constructing the synthetic networks (see Methods). The value for burstiness measured in the synthetic networks follows the same trends as the analytical solution, although some deviations exist because of the use of edge lifespans in constructing the synthetic networks. In particular, the deviations likely result from the fact that the broad distribution of edge lifespans in general reduces the probability of large edge IETs. The parabolic shape arises from the non-monotonic behaviour of the coefficient of variation (i.e., the ratio of the standard deviation *σ* to the mean *m*) as *β* is increased. From Eq. 5, we can see that *B*_*E*_ is a monotonically increasing function of *σ*/*m*. As *β* is increased, both *σ* and *m* decrease, but their ratio first increases and then decreases.

## Discussion

Our simulations on empirical networks with node turnover show that PTN can yield dramatically different predictions of spreading speed compared to PT. For nodes that have lifespans shorter than the sampling time, PT can increase the lifespan. The mean interevent time of the node thus increases. This results in a lower number of contacts per unit time, impeding the spreading ability of that node. A PT reference network could therefore exhibit slower spreading dynamics than the original network simply because of the increased mean interevent time of each node. We see this potentially spurious effect in the Prostitution^22^ and Ant^9^ networks, both of which have signatures of node turnover (cf. Fig. 2). In particular, individuals in the Prostitution network enter and leave the network at various times during the sampling period, while individuals in the Ant network enter the network at different times but stay until the end of sampling.

In the synthetic networks, spreading on PT models is faster on average than on PTE models, and the differences in spreading speed between the PT and PTE reference models increases with the heterogeneity of edge lifespans. As with nodes, the spreading ability of an edge is highly dependent on its lifespan. For an edge with a short lifespan, contacts on that edge are constrained to a short time window. In contrast, when the same number of contacts is dispersed over an edge with a long lifespan, there is a higher potential for the edge to contribute to spreading. The PT randomization generally increases the lifespan of edges and dispersion of contacts on edges. This explains why PT shows faster average spreading dynamics compared to PTE for our synthetic networks. Our results are thus consistent with the notion that temporal sparsity can slow down spreading^29^. Similar to the synthetic networks, for the two empirical networks without node turnover (Conference and Workplace), the PT model also exhibited faster average spreading dynamics than PTE.

On the other hand, the situation is more complicated in the empirical networks with node turnover, as the increased spreading speed induced by longer edge lifespans and the decreased speed due to longer node interevent times together determine the spreading dynamics on the PT model. In the Prostitution network, most edges have a single contact and PT cannot extend their lifespan, so the damping effect of longer node interevent times dominates; this explains the slower spreading on PT in Fig. 4(B). In the Ant network, however, these opposing effects coexist, resulting in the intersecting behavior in Fig. 4(A). In particular, spreading on PT is slower in the short-time limit because of the larger mean node IETs, while the longer edge lifespans enhance the long-time spreading behavior (which is also observed in the two face-to-face networks, see Fig. 4(C,D)).

While many studies have explored the effect of burstiness on spreading dynamics using the PT reference model^21,22,30,31^, only a few studies have examined the influence of node and edge lifetimes^16,24^. Rocha and Blondel^23^ used synthetic networks to show that node turnover can enhance spreading when long node IETs are removed. Holme and Liljeros^16^ use spreading simulations on several specially-constructed reference networks to demonstrate that the steady state of epidemic spreading – represented by outbreak size – is mainly determined by the birth and death of links and the total number of contacts over links. In contrast, our study focuses on the transient spreading dynamics, i.e., the speed-up or slow-down of spreading in the short-time limit, and how they are affected by different temporal structures. Like PTE, the reference models used in their study of epidemic outbreaks preserve the lifespan of edges. However, unlike PTE, they do not preserve periodic temporal patterns. This makes it impossible to separate the effects of bursty edge IETs from the effects of daily patterns.

In summary, we have shown that the choice of reference model can lead to dramatically different predictions of spreading dynamics on a temporal network. We used four empirical networks to highlight the effects of node turnover, and synthetic networks to study the effect of edge lifespans on spreading dynamics. Network researchers typically use reference models to draw broad conclusions about how different temporal features of a given network affect spreading dynamics on the network. Since these conclusions are all framed relative to the reference model, it is extremely important to choose an appropriate model. Our results highlight that for networks with heterogeneous node or edge lifespans one must carefully consider which model is best suited to answer the question about spreading dynamics.

Given these observations, one naturally wonders how to choose whether or not to preserve node or edge lifetimes when creating reference models. Our results appear to suggest that if domain knowledge – or some metric for capturing the continuous presence of nodes – indicates that nodes with a short network-lifespan were inactive before or after the network was being sampled, then the PTN reference model should be used. Otherwise, the established PT reference model might be a better choice. As an example, in the case of the honey bee social networks (Gernat et al., under review (2017)), the times of birth and death of each individual are known. Since these correspond to the first and last times when an individual could participate in an interaction, it is more natural to apply PTN than PT in this case. Analogously, in proximity networks constructed for spatially-segregated individuals, domain knowledge might suggest that there is link turnover as some individuals move from one spatial location to another. In such a situation, it may be prudent to apply the PTE model rather than PT.

## Methods

### Empirical networks

The Ant data set corresponds to Ant colony “1-1” from ref.^9^. Although the original data set consists of directed interactions, for consistency with the other data sets, we ignore directionality and construct undirected networks. Note that multiple edges at one timestamp may occur after simplification because two directed links between a pair of nodes could be reduced to a single link after link directions are ignored; in such cases, we further condense the multiple edges to a single edge in our analysis.

For the Prostitution network, we follow the processing steps in ref.^22^, i.e., we ignore the first 1,000 days of the experiment. The time resolution (shortest time between two contacts) is one day in the Prostitution data sets, even though multiple interactions between two nodes can happen within one day. We simply regard these multiple contacts as a single contact in our analysis.

While the Ant data set consists of a sequence of interactions, the proximity data sets consist of a sequence of contacts, from which we inferred a sequence of interactions. Each set of contacts on an edge with inter-contact times equal to the temporal resolution of the experiment are condensed into a single interaction. We use the start time of these interactions in our spreading simulations.

### Generation of structure-preserving randomized reference models

#### PTE reference model

The idea underlying the PTE reference model is as follows: consider two edges *ij* and *lm*, each of which has a sequence of contacts times ${{\mathscr{T}}}_{ij}$ and ${{\mathscr{T}}}_{lm}$. Let ${t}_{ij}^{s}$ and ${t}_{ij}^{f}$ be the minimum and maximum times in ${{\mathscr{T}}}_{ij}$ (and similarly for edge *lm*). Then, two timestamps ${t}_{1}\in {{\mathscr{T}}}_{ij}$ and ${t}_{2}\in {{\mathscr{T}}}_{lm}$ can be permuted if and only if two conditions are satisfied: (1) ${t}_{1},\,{t}_{2}\in ({\rm{\max }}\,\{{t}_{ij}^{s},{t}_{lm}^{s}\},\,{\rm{\min }}\,\{{t}_{ij}^{f},{t}_{lm}^{f}\})$ and (2) ${t}_{1}\notin {{\mathscr{T}}}_{lm}$ and ${t}_{2}\notin {{\mathscr{T}}}_{ij}$. Condition (1) ensures that the times of the first and last edge events are preserved in randomization, and thus the lifespans and mean IETs of edges are preserved. Condition (2) prevents multiple contacts that take place at the same time from being assigned to the same edge. Such contacts are to be avoided because they effectively reduce to a single contact, and so a contact is essentially lost upon randomization. When applying the PTE model to generate a reference network, we set the maximum number of permutation attempts for each permutation to *I*_max_ = 100. If a contact cannot be permuted after *I*_max_ attempts, it is skipped. In our simulations, fewer than 1% of the contacts were skipped.

#### PTN reference model

The construction of a PTN reference network is rather similar to that for PTE. This time, let ${{\mathscr{T}}}_{i}$ represent the set of contact times for a node *i*, and let ${t}_{i}^{s}=\,{\rm{\min }}\,{{\mathscr{T}}}_{i}$ and ${t}_{i}^{f}=\,{\rm{\max }}\,{{\mathscr{T}}}_{i}$ be the first and last contact times. Then two timestamps, *t*_1_ from edge *ij* and *t*_2_ from edge *lm*, can be permuted if and only if: (1) ${t}_{1},\,{t}_{2}\in ({\rm{\max }}\,\{{t}_{i}^{s},{t}_{j}^{s},{t}_{l}^{s},{t}_{m}^{s}\},\,{\rm{\min }}\,\{{t}_{i}^{f},{t}_{j}^{f},{t}_{l}^{f},{t}_{m}^{f}\})$ and (2) ${t}_{1}\notin {{\mathscr{T}}}_{lm}$ and ${t}_{2}\notin {{\mathscr{T}}}_{ij}$. Similar to PTE, we set the maximum number of permutation attempts for a given contact to *I*_max_ = 100. We expect the fraction of skipped contacts for nodes to be less than that for edges, because PTE is more constrained than PTN. This makes it more likely that permutations of contacts in PTN are accepted, because nodes generally have longer lifespan than their associated edges.

### Spreading model

To study the spreading properties of the synthetic and empirical temporal networks, as well as the corresponding reference models, we use the simplest compartmental model of epidemic spreading, namely the deterministic susceptible-infected (SI) model. In this model, each node is in one of two states – susceptible (S) or infected (I) – and an infected node always infects a susceptible node when they come in contact. In each simulation run, an initial interaction is chosen uniformly at random, and the two nodes involved are set to the infected state. During the simulation of the SI model, we measure the prevalence *I*(*t*)/*N* (i.e., fraction of infected nodes) as a function of the time *t* since the initial infection. Since we are interested in studying the effects of node/edge lifespans within the sampling interval, we do not use periodic boundary conditions, which would distort those lifespans. Instead, we ensure that the time of initial infection is early enough so that most nodes are infected by the end of the sampling time *T*. Specifically, we choose initial interactions with timestamps in the range [*t*_0_, *t*_0_ + *μT*], where *t*_0_ is the sampling start time and *μ* < 1. Based on trial simulations, we chose *μ* = 0.4, 0.3, 0.9, 0.8 for the Ant, Prostitution, Conference and Workplace networks, respectively. These values ensure that most nodes are infected within a time (1 − *μ*)*T* after the initial infection. For each network, we repeat the SI simulation 500 times with different initial conditions and calculate the average prevalence 〈*I*(*t*)/*N*〉 as a function of time. For each of the reference models, we average the result over four reference networks.

### Construction of synthetic networks

Our method of constructing synthetic temporal networks is based on the generative model developed by Holme^6,32^. In this generative model, a temporal network is constructed in two stages: (1) the static network structure is generated, and (2) each edge of the static network is assigned a sequence of contacts to make it a temporal network.

Here, we preserve the first two steps of the protocol described in ref.^6^, but we modify the rest of the protocol to avoid potential complications introduced by the rescaling process in ref.^6^. We assign to each edge a temporal sequence of contacts in the time interval [*t*_*b*_, *t*_*b*_ + *τ*] during which the edge is active, where *t*_*b*_ denotes the start time of the active interval and *τ* represents the lifespan of that edge. Next, we assign a sequence of times within this active interval. Below, we describe the construction process in detail.

#### **Step** (**i**)

Construct the topology for a network with *N* nodes using the Erdős-Rényi model *G*(*N*, *p*).

#### **Step** (**ii**)

Sample edge lifespans *τ* from the following probability distribution:

3 $$P(\tau ;\alpha ,{\tau }_{{\rm{\max }}},\lambda )\sim \{\begin{array}{c}{\tau }^{-\alpha }\\ 0\end{array}\,\begin{array}{l}{\rm{if}}\,\tau \in [\lambda {\tau }_{{\rm{\max }}},{\tau }_{{\rm{\max }}}]\\ {\rm{otherwise}}\end{array}$$

where *τ*_max_ is the maximum active interval of edges, and *λ* represent the ratio of minimum to maximum of active intervals (*λ* ≤ 1). By adjusting *λ*, one can control the heterogeneity of edge lifespans. In particular, we increase the heterogeneity by decreasing *λ*. In the limiting case *λ* = 1, networks have homogeneous distribution of edge lifespans.

#### **Step** (**iii**)

Choose the start time *t*_*b*_ of the active interval for each edge uniformly at random from [0, *T* − *τ*]. Since the start times are different across edges, the parameter *λ* defined in Step (ii) tunes the extent to which the active intervals of different edges overlap. At higher *λ*, the overlap increases. For *λ* = 1 and *τ*_max_ = *T*, the active intervals of all edges overlap completely.

#### **Step** (**iv**)

Assign a sequence of contact times for each edge by sampling a set of interevent times {Δ_*i*_} from the following truncated power-law distribution:

4 $$P({\rm{\Delta }};\beta ,{{\rm{\Delta }}}_{{\rm{\min }}},{{\rm{\Delta }}}_{{\rm{\max }}})\sim \{\begin{array}{c}{{\rm{\Delta }}}^{-\beta }\\ 0\end{array}\,\begin{array}{l}{\rm{if}}\,{\rm{\Delta }}\in [{{\rm{\Delta }}}_{{\rm{\min }}},{{\rm{\Delta }}}_{{\rm{\max }}}]\\ {\rm{otherwise}}\end{array}$$

The sequence of contact times is then {*t*_*b*_, *t*_*b*_ + Δ_1_, *t*_*b*_ + Δ_1_ + Δ_2_, *t*_*b*_ + Δ_1_ + Δ_2_ + Δ_3_, …}. However, we only retain the subset of these contact times that fall within the active interval [*t*_*b*_, *t*_*b*_ + *τ*] of an edge. For the analyses reported here, we chose Δ_min_ = 1 and *τ*_max_ = Δ_max_ = *T*.

We chose the parameter values based on empirical data sets; in particular, based on the values for the data set in ref.^18^, we set *α* = 0.8 and *λ* = 10^−2^. The range for the IETs in these data sets spans 4–5 orders of magnitude, so we chose Δ_max_ = 10^4^. The power-law exponent *β* of the IET distribution ranges from 1.0 to 1.6 in refs^33,34^ and we set it to 1.2 unless otherwise stated.

### Burstiness characterization

A random (Poissonian) temporal process gives rise to an exponential distribution of interevent times. The burstiness coefficient^28^ is used to quantify the deviation of a given time series from a Poissonian signal, and is based on measuring the extent to which the coefficient of variation deviates from unity. In order to avoid finite-size effects in measuring the burstiness parameter^6,35^, we collect the IETs from all nodes/edges and measure burstiness for this aggregated sequence of IETs. For a sequence of IETs with mean *m* and standard deviation *σ*, the burstiness coefficient is defined as

5 $$B\equiv \frac{\sigma /m-1}{\sigma /m+1}.$$

For the IET distribution in Eq. 4, we can calculate *m* and *σ* analytically:

6 $$m=\frac{1-\beta }{2-\beta }(\frac{{{\rm{\Delta }}}_{{\rm{\max }}}^{2-\beta }-{{\rm{\Delta }}}_{{\rm{\min }}}^{2-\beta }}{{{\rm{\Delta }}}_{{\rm{\max }}}^{1-\beta }-{{\rm{\Delta }}}_{{\rm{\min }}}^{1-\beta }})$$

and

7 $$\sigma =\sqrt{\hat{m}-{m}^{2}},$$

in terms of the second moment of the distribution

8 $$\hat{m}=\frac{1-\beta }{3-\beta }(\frac{{{\rm{\Delta }}}_{{\rm{\max }}}^{3-\beta }-{{\rm{\Delta }}}_{{\rm{\min }}}^{3-\beta }}{{{\rm{\Delta }}}_{{\rm{\max }}}^{1-\beta }-{{\rm{\Delta }}}_{{\rm{\min }}}^{1-\beta }}),$$

assuming that $$\beta \notin \{1,2,3\}$$.
